# Huanglian Jiedu Decoction in the Treatment of the Traditional Chinese Medicine Syndrome “Shanghuo”–An Intervention Study

**DOI:** 10.3389/fphar.2021.616318

**Published:** 2021-04-30

**Authors:** Keke Luo, Haiyu Zhao, Baolin Bian, Xiaolu Wei, Nan Si, Adelheid Brantner, Xiaorui Fan, Xinru Gu, Yanyan Zhou, Hongjie Wang

**Affiliations:** ^1^ Institute of Chinese Materia Medica, China Academy of Chinese Medical Sciences, Beijing, China; ^2^ Institute of Pharmaceutical Sciences, University of Graz, Graz, Austria

**Keywords:** Shanghuo, huanglian jiedu decoction, biomarkers, inflammatory factors, oxidative stress and energy metabolism factors, omics analysis, network analysis

## Abstract

“Shanghuo” (“excessive internal heat”) is caused by exuberant endogenous fire, which does not have a comprehensive and systematic traditional Chinese medicine theory. In previous study, we had evaluated the therapeutic effect of Huanglian Jiedu Decoction (HLJDD) (granule) on patients with “Shanghuo”, however, the specific mechanism was not clear, which need further exploration. To explain its intervention mechanism, we select 57 patients with oral diseases caused by “Shanghuo” and 20 health volunteers to divide into oral disease group, HLJDD intervention group and healthy control group. Firstly, biochemical indicators before and after HLJDD intervention are detected, such as inflammatory factors, oxidative stress factors and energy metabolism factors. The results exhibit that HLJDD significantly decreases indicators succinic acid (*p* < 0.001); tumor necrosis factor-alpha, adenosine triphosphate, citric acid (*p* < 0.01); interleukin-8 (IL-8), 4-hydroxynonenal, pyruvic acid, lactate dehydrogenase (*p* < 0.05). The levels of glucocorticoid, adrenocorticotropic hormone (*p* < 0.01); lactic acid, IL-4, IL-10 (*p* < 0.05) significantly increase after HLJDD intervention. In addition, we adopt multi-omics analysis approach to investigate the potential biomarkers. Nontargeted metabolomics demonstrate that the levels of 7 differential metabolites approach that in the healthy control group after HLJDD intervention, which are correlated with histidine metabolism, beta-alanine metabolism and sphingolipid metabolism through metabolic pathway analysis. Targeted lipidomics results and receiver operating characteristic curve analysis show that 13 differential lipids are identified in the three groups mainly focuse on lysophosphatidylcholines, lysophosphatidylethanolamines. Finally, the network associations of those differential biomarkers reveal the regulation of adenosine triphosphate and tricarboxylic acid cycle play essential role in the therapeutic effect mechanism of HLJDD in “Shanghuo”. The study has laid the foundation for further revealing the mechanism and finding clinical biomarkers related to “Shanghuo”.

## Introduction

“Shanghuo” (“excessive internal heat”) is a traditional Chinese medicine (TCM) terminology, and a common name for symptoms including fever, thirst with desire to drink liquids, constipation, sparse yellow urine, a dry and reddened tongue with a yellow coating, and a rapid or surging pulse (Liu et al., 2014). The term “Shanghuo” indicates an imbalance of Yin and Yang in the human body caused by “exuberant endogenous fire”. The main symptoms are redness, swelling, fever, and pain in the skin and mucous membranes of the head and face. In modern medicine, many factors, including mental stress, overwork, dietary irritation, oral microbiome disorders, and immunological abnormalities are related to the appearance of “Shanghuo” (Zheng et al., 2017; Du et al., 2018; Lei et al., 2018). For example, the pathogenesis of foodborne “Shanghuo” is mostly related to the composition of the food itself. An appropriate amount of food intake would not cause the body to develop “Shanghuo”. However, excessive intake could affect the body's neuroendocrine system, immune system, etc. to disrupt the balance of Yin and Yang in the body, and a series of “Shanghuo” symptoms would appear (Zhang and Jiang, 2020). For example, overconsumption of Korean red ginseng could induce “Shanghuo”, which has a close relationship with an accelerated tricarboxylic acid (TCA) cycle and increased adenosine monophosphate (AMP)-activated protein kinase (AMPK) activity (Zhao et al., 2020). “Shanghuo” has a high incidence and is related to the onset and evolution of diseases such as gingivitis, recurrent oral ulceration (ROU) or recurrent aphthous stomatitis. With the shift in the medical model toward preventive medicine, there is an urgent need to investigate the onset and prevention of “Shanghuo”.

Some studies have emphasized the importance of TCM treatment of “Shanghuo” in maintaining normal physiological functions, such as immunity, lipid metabolism and reactive oxygen species (ROS) clearance (Pan et al., 2020). Huanglian Jiedu Decoction (HLJDD) was originally contained in Zhouhou Beiji Prescription and consisted of *Rhizoma Coptidis*, *Radix Scutellariae*, *Cortex Phellodendri*, and *Fructus Gardeniae* at a ratio of 3:2:2:3. It is a classic Chinese herbal prescription that has attracted substantial attention from researchers. HLJDD is a typical syndrome drug widely used in heat clearing and detoxifying. The main therapeutic effects are purging fire for removing toxins and it is used for treating all kinds of heat toxins and San Jiao heat syndromes. Modern research studies have confirmed that the potential applications of HLJDD have increased, including anti-inflammatory, antioxidant, antithrombotic, hypoglycemic, hypolipidemic, hypotensive, and antitumoral effects. (Fang, 2015; Gao et al., 2018; Gu et al., 2018; Hao and He, 2018). The “Four-nature Theory” divides all Chinese herbs into four categories, including “cold” “hot” “warm” and “cool” herbs. According to this theory, the four herbs in HLJDD are all considered “heat clearing” herbs, which means that they all have the ability to remove “body fire”. The symptoms show up we often call it “Shanghuo”. The symptoms of redness, swelling, heat, and pain manifest in “Shanghuo” and inflammation and some clinical manifestations of “Shanghuo” were due to the destruction of the balance between anti-inflammatory and proinflammatory processes (Ge et al., 2011). Pharmacokinetic studies have found that HLJDD synergistically inhibits inflammation through its three major active ingredients (iridoids, alkaloids and flavonoids) (Ren et al., 2016). However, to date, the underlying mechanism of HLJDD in treating “Shanghuo” is still a mystery and needs further exploration.

In our previous research, high performance liquid chromatography-Q-Exactive and ultra performance liquid chromatography-QqQ mass spectrum (MS) analytical techniques were applied for qualitative and quantitative analysis of the chemical profiles of HLJDD. Sixty-nine compounds were identified, including iridoids, alkaloids, flavonoids, triterpenoids, monoterpenes and phenolic acids, and 17 major characteristic constituents were selected as quality control markers in HLJDD (Yang et al., 2013). Combined with the research on the original form of HLJDD and its metabolites in rat plasma (Zuo et al., 2014), these results clarified the possible material basis of the *in vivo* effects exerted by HLJDD and provided basic chemical information for our study. Recently, scholars evaluated the effects of HLJDD by simulating rat “Shanghuo” gingivitis, and the results showed that HLJDD inhibited energy metabolism and oxidative stress through tuberous sclerosis complex signaling pathway and glycolysis-related molecules (Zhang et al., 2017). The active ingredients in HLJDD interfered with inflammation by regulating intracellular free radicals, cholesterol, and mitochondrial ion homeostasis (Liu et al., 2019). Through clinical research, it was confirmed that HLJDD (granule) significantly repaired endogenous small molecule metabolic disorders in the urine of patients with stomatological diseases caused by “the exuberance of stomach fire”. (Zhou et al., 2019). Combined with an extensive literature search, in the serum metabolic profile of the “Shanghuo” group, a variety of lipids and bilirubin decreased to a certain extent (Wu et al., 2018). As a physiological antioxidant, bilirubin not only inhibited lipid oxidation and ROS formation but also suppressed immune and inflammatory reactions (Rizzo et al., 2010; Vogel and Zucker, 2016). The interaction of biomolecules (such as phospholipids) with oxidants in cells can cause cellular dysfunction, which in turn can lead to cell death. Molecules formed during oxidation could be used as biomarkers to quantify oxidative stress in the human body (Syslová et al., 2014). Some scholars believe that AMPK function is inhibited in “Shanghuo” (Zhao et al., 2018). The AMPK cascade not only regulates the energy balance in the body, but also regulates inflammation in two ways (Tang et al., 2011). In summary, the occurrence of “Shanghuo” is closely related to inflammatory immunity, oxidative stress, energy metabolism and their interactions.

In this study, we investigated the intervention mechanism and potential biomarkers of HLJDD (granule) in the treatment of “Shanghuo”. First, we decided to detect the changes in related biochemical indicators before and after HLJDD intervention, and multiplex cytokine assay technology was used to detect inflammatory factors interleukin-4 (IL-4), IL-1β, IL-8, IL-10, IL-6, IL-12p70, IL-13, monocyte chemotactic protein-1 (MCP-1), macrophage inflammatory protein-1alpha (MIP-1α), and tumor necrosis factor-alpha (TNF-α). Then we analyzed oxidative stress factors extracellular superoxide dismutase (SOD3), 4-hydroxynonenal (4-HNE) and adrenocorticotropic hormone (ACTH) and energy metabolism factors citric acid (CA), succinic acid (SA), glucocorticoid (GC), adenosine triphosphate (ATP), lactate dehydrogenase (LDH), lactic acid (LA), pyruvic acid (PA), thyroid-stimulating hormone (TSH) using ELISA technology. Additionally, metabolomics helped clarify the course of the disease and provided useful information for preventing and diagnosing disease, discussing pathogenesis, and researching drug action mechanisms (Liu et al., 2015; Chen et al., 2016; Hu et al., 2016). In recent years, targeted and untargeted metabolomics have been widely used to comprehensively study chemical variation. The advantage of non-target metabolomics is to qualitatively determine all measurable analytes in a sample, including chemical unknowns, while target metabolomics further performs absolute quantification on a set of defined chemical substances. By providing comprehensively qualitative and quantitative information on total secondary metabolites, the integration of target metabolites and non-target metabolite groups provides deeper insights into processing chemistry (Liang et al., 2018). To explore potential plasma biomarkers from “Shanghuo” patients, we adopted nontargeted metabolomics and targeted lipidomics approaches using Q-Exactive MS and QqQ MS, and our results might play an important role in exploring and revealing potential biomarkers. Finally, the network associations of the differentially expressed biomarkers were explored through MetaboAnalyst and Cytoscape v3.4.0 software from three perspectives, including the KEGG global metabolic network, metabolite-metabolite interaction network and protein-enzyme/transporter-metabolite network. Our study provides a new experimental basis to understand the treatment mechanism of HLJDD in “Shanghuo” and proposes potential biomarkers and metabolic pathways for “Shanghuo” diagnosis. This study might lay the foundation for further understanding the pathogenesis of “Shanghuo” and the intervention mechanism of HLJDD in “Shanghuo”.

## Materials and Methods

### Study Population

Cases were collected from January 2018 to August 2018 in Beijing University of Chinese Medicine Third Affiliated Hospital, Dongfang Hospital (Beijing University of Chinese Medicine), and the Department of Stomatology of Affiliated Hospital of Chengdu University of TCM. According to the inclusion and exclusion criteria of TCM syndromes and clinical manifestations in combination with western medicine diagnosis of “Shanghuo”, a total of 57 “Shanghuo” cases were included as the oral disease group. The detailed information of the diagnostic criteria is listed in the supplement materials. The cases we selected were diagnosed with recurrent oral ulcers, pericyxitis of wisdom teeth, and recurrent herpes stomatitis caused by exuberance of stomach fire. The same 57 patients were also treated with HLJDD (granule) as the intervention group. The average age of the patients was 36.22 ± 13.26 years, and the group included 21 males and 36 females aged 21–60 years. At the same time, we recruited 20 healthy volunteers as the healthy control group, which consisted of 6 males and 14 females. Their ages ranged from 22 to 58 years, with an average age of 33.30 ± 13.33 years.

### Preparation of Huanglian Jiedu Decoction Formula Granule

This formula granule was composed of *Rhizoma Coptidis* 900 g, *Radix Scutellariae* 600 g, *Cortex Phellodendri* 600 g, and *Fructus Gardeniae* 900 g. These four drugs were identified by taxonomy, namely *Coptis chinensis* Franch.*, Scutellaria baicalensis* Georgi*, Phellodendron chinense* Schneid. and *Gardenia jasminoides* Ellis. The above four Chinese medicines were soaked in water for 1 h and decocted twice, then filtered, and the filtrate was combined and concentrated into a thick paste, dried under reduced pressure, and pulverized into powder. Mixed evenly according to the ratio of 60 g of microcrystalline cellulose per 100 g of powder, and made into HLJDD formula granule (3.2 g/bag). They were provided by Institute of Chinese Materia Medica, China Academy of Chinese Medical Sciences. The qualitative analysis of the granules and the quantitative analysis of its 9 main active ingredients (coptisine hydrochloride, epiberberine, berberine, palmatine, baicalein, baicalin, wogonoside, genipin 1-gentiobioside, and geniposide) are shown in the supplementary materials.

### Dosage of Huanglian Jiedu Decoction Formula Granules

Patients with oral diseases were given HLJDD formula granules within 0.5–1 h after breakfast and dinner, mixed with hot water above 80 °C and taken 1 bag at a time, twice daily. The course of treatment was 3–5 days until the main symptoms disappeared.

### Chemicals and Reagents

HLJDD granules (3.2 g/bag) were provided by Institute of Chinese Materia Medica, China Academy of Chinese Medical Sciences (Batch number: 1801001Y). Methanol, formic acid, water and acetonitrile were purchased from Thermo Fisher Scientific (China) Co., Ltd (Batch numbers: 174,486, 168,642, 175,160). Ammonium acetate was obtained from Sigma-Aldrich. All other chemicals and solvents were of analytical grade.

### Luminex Multiplex Cytokine Assay of Inflammatory Factors

To explore the changes in biochemical indicators of inflammation before and after HLJDD intervention, the levels of 10 inflammatory factors in plasma were measured. The factors included interleukin-4 (IL-4), IL-1β, IL-8, IL-10, IL-6,IL-12p70, IL-13, monocyte chemotactic protein-1 (MCP-1), macrophage inflammatory protein-1 alpha (MIP-1α) and tumor necrosis factor-α (TNF-α). The concentrations (pg/ml) of cytokines in plasma were analyzed by Luminex Multiplex Cytokine Kits (Panomics/Affymetrix, Santa Clara,CA) according to the manufacturer’s recommendation.

### Enzyme-Linked Immunosorbent Assay of Oxidative Stress and Energy Metabolism Factors

To analyze the effects of HLJDD on energy metabolism and oxidative stress in the “Shanghuo” group, we measured citric acid (CA), succinic acid (SA), glucocorticoid (GC), adenosine triphosphate (ATP), lactate dehydrogenase (LDH), lactic acid (LA), pyruvic acid (PA), thyroid-stimulating hormone (TSH), extracellular superoxide dismutase (SOD3), 4-hydroxynonenal (4-HNE) and adrenocorticotropic hormone (ACTH) in plasma by ELISA method. All operations were performed strictly in accordance with the corresponding manufacturer’s instructions for ELISA (BioAssay Systems, United States).

### Nontargeted Metabolomics Using UPLC Q-Exactive MS

Plasma samples were thawed on ice at 4°C for 30–60 min. One hundred microliters of each plasma sample was transferred to a 1.5 ml centrifuge tube containing 300 µL acetonitrile. Then the sample mixture was vortexed for 15 s and sonicated for 3 min. After centrifugation (12,000 rpm, 4°C, 5 min), 100 µL of the supernatant was transferred into standard autosampler vials with 250 µL microinlets.

Analysis was performed on a Thermo Scientific™ Dionex™ UltiMate™ 3,000 Rapid Separation LC system coupled to a Q Exactive™ hybrid quadrupole Orbitrap mass spectrometer equipped with a HESI-II probe. The column was a BEH amide column (2.1 × 100 mm, 1.7 µm, Waters) operated at 40°C. For HILIC separation, mobile phase A was acetonitrile, mobile phase B was water, and both A and B contained 0.1% formic acid and 10 mmol/L ammonium acetate. The sample manager temperature was set at 4°C, the flow rate was 300 μL/min and the injection volume was 1 µL. Then the samples were eluted using the following gradient conditions: 0–1 min, 5% B; 1–7 min, 5–50% B; 7–9 min, 50% B; 9–9.1 min, 95% B, and the equilibration time was 3.9 min with 95% B.

The positive HESI-II spray voltage was 3.7 kV, the heated capillary temperature was 320°C, the sheath gas pressure was 30 psi, the auxiliary gas setting was 10 psi, and the heated vaporizer temperature was 300°C. A full scan range from 50 to 1,500 (m/z) was used. The resolution was set at 70,000. The maximum isolation time was 50 ms. Automated gain control was targeted at 1 × 10^6^ ions. The LC-MS system was controlled using Xcalibur 2.2 SP1.48 software (Thermo Fisher Scientific).

### Targeted Lipidomics Using UPLC QqQ MS

Plasma samples were thawed on ice at 4°C for 30–60 min. Then, 600 µL of methanol/chloroform (1:3) organic solvent was added to a labeled 1.5 ml centrifuge tube containing 100 µL of plasma sample with 10 µL of lipid internal standard solution. The samples were then sonicated for 1 h and mixed well. A volume of 100 µL of water was added to each sample, followed by centrifugation at 12,000 rpm at 4 °C for 10 min. A volume of 300 µL of the chloroform layer was collected, concentrated and dried, after which 200 µL of isopropanol/acetonitrile (1/1) was added for reconstitution. The samples were then sonicated and centrifuged at 12,000 rpm at 4°C for 10 min.

The supernatant was then analyzed by LC-20AXR Ultra-Fast Liquid Chromatography coupled to a Qtrap 5,500 tandem quadrupole mass spectrometer. A Waters UPLC BEH C8 (1.7 µm, 2.1 mm × 100 mm) was used for the separation of lipids; acetonitrile/water (60/40) was used for mobile phase A and isopropanol/acetonitrile (90/10) was used for mobile phase B. Both the A and B phases contained 5 mmol/L ammonium acetate. The samples were eluted using the following gradient conditions: 0–2 min, 0–30% B; 2–12 min, 30–70% B; 12–12.5 min, 70–95% B; 12.5–13 min, 95–100% B; 13–13.1 min, 100–0% B, and the equilibration time was 1.9 min with 0% B. The column was operated at 40°C, the flow rate was 260 μl/min and the sample manager temperature was set at 4°C.

The electrospray ionization source was operated in positive ionization mode with the following settings: spray voltage, 5.5 kV; heated capillary temperature, 500°C; sheath gas pressure, 30 psi; auxiliary gas, 10 psi; and heated vaporizer temperature, 300°C. The analytes were determined by monitoring the precursor–product transition in MRM mode.

### Quality Control Evaluation Method

To monitor the system’s stability and the reproducibility of the samples, quality control (QC) samples were prepared by pooling equal volumes of each plasma sample. The pretreatment of the QC samples was in accordance with that of the plasma samples. The QC samples had the same composition and should have clustered together. Relatively clustered QC samples demonstrate that the system has good repeatability and the collected data are worthy of further study. In our study, three QC samples were continuously injected at the beginning of the run and then injected at regular intervals of six or eight samples throughout the analytical run to provide data from which repeatability could be assessed.

### Data Processing and Statistical Analysis

For nontargeted metabolomics, all data obtained were processed using Progenesis QI software for imputing raw data, aligning and picking peaks and normalization to produce peak intensities for retention time (t_R_) and m/z data pairs. Targeted lipidomics data were processed using Skyline software for the relative quantification of the lipid species according to the retention time (t_R_) and accurate mass in the constructed lipid database. Then, the adduct ions of each “feature” (m/z, t_R_) were deconvoluted, and these features were identified in the human metabolome database and lipidmaps. Preprocessed data were imported to SIMCA 14.1 software (Umetrics) for multivariate analysis. Unsupervized separation was assessed by principal component analysis (PCA) using Parteo standardization to distinguish the differences in samples among all variables. The data were further processed by orthogonal partial least squares discrimination analysis (OPLS-DA) to identify the potential biomarkers based on the variable importance in the projection (VIP) value (threshold value >1) and *t*-test (*p* < 0.05).

The differences of biochemical indicators between two groups were analyzed by Wilcoxon matched-pairs signed rank test using GraphPad Prism version 8.01. All data are presented as the mean ± SD/SEM. In all cases, a value of *p* < 0.05 was considered significant. In addition, we use the ROC curve drawing function in the GraphPad prism version 8.01 software to evaluate the plasma targeted lipid analysis results. When the area under the curve is greater than 0.7, we believed that these indicators have a certain degree of accuracy for the diagnosis of the disease. The network associations of differentially expressed potential biomarkers were explored from three perspectives, including the KEGG global metabolic network made by MetaboAnalyst software, metabolite-metabolite interaction network and protein-enzyme/transporter-metabolite network produced by Cytoscape v3.4.0 software.

## Results

### Inflammatory Factors

**FIGURE 1:**
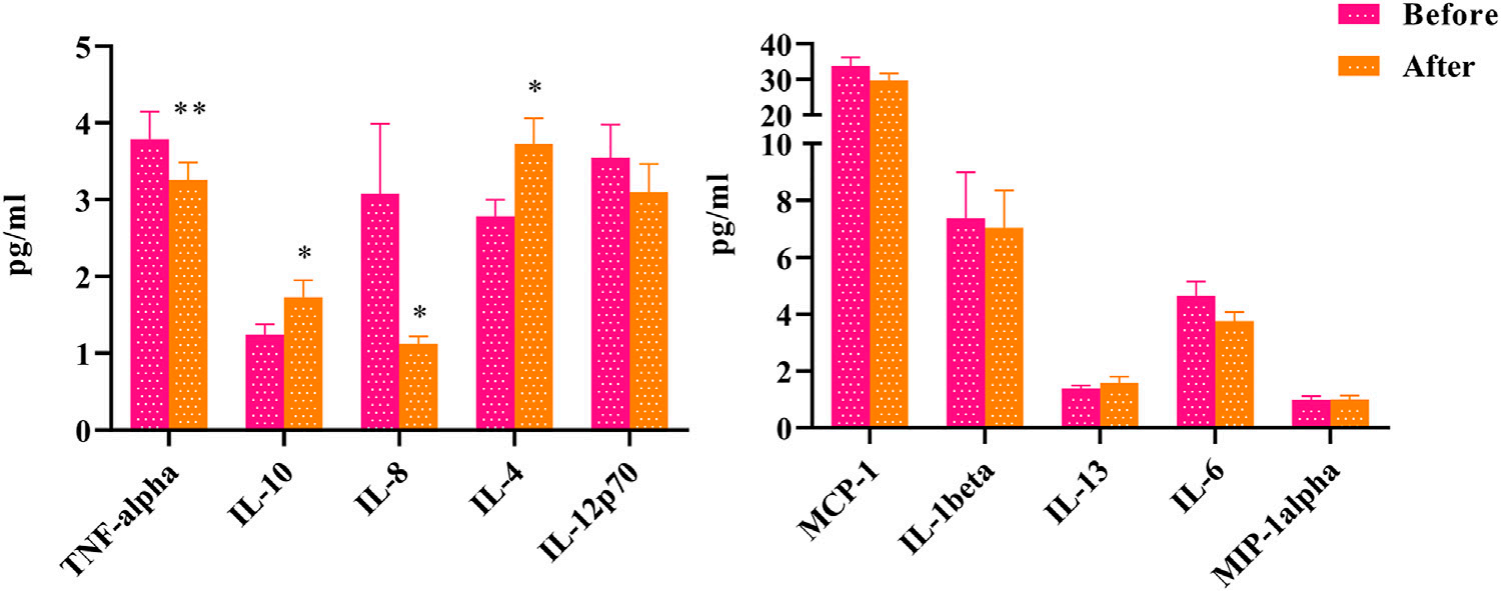
The changes of inflammatory factors before and after HLJDD intervention (Before) Oral disease group (After) HLJDD intervention group (*p < 0.05, **p < 0.01).

To explore the clinical effect of HLJDD on inflammation in “Shanghuo”, we had assayed the levels of 10 cytokines and chemokines in plasma before and after HLJDD intervention. Compared with the oral disease group, the HLJDD intervention group showed significantly reduced levels of TNF-α (*p* < 0.01) and IL-8 (*p* < 0.05). The levels of IL-4 and IL-10 were significantly increased after HLJDD intervention (*p* < 0.05). In addition, the levels of IL-6, IL-1β, IL-12p70, and MCP-1 showed downward trends, and the levels of IL-13 and MIP-1 showed upward trends, but these differences were not significant (Figure 1).

### Energy Metabolism

**FIGURE 2:**
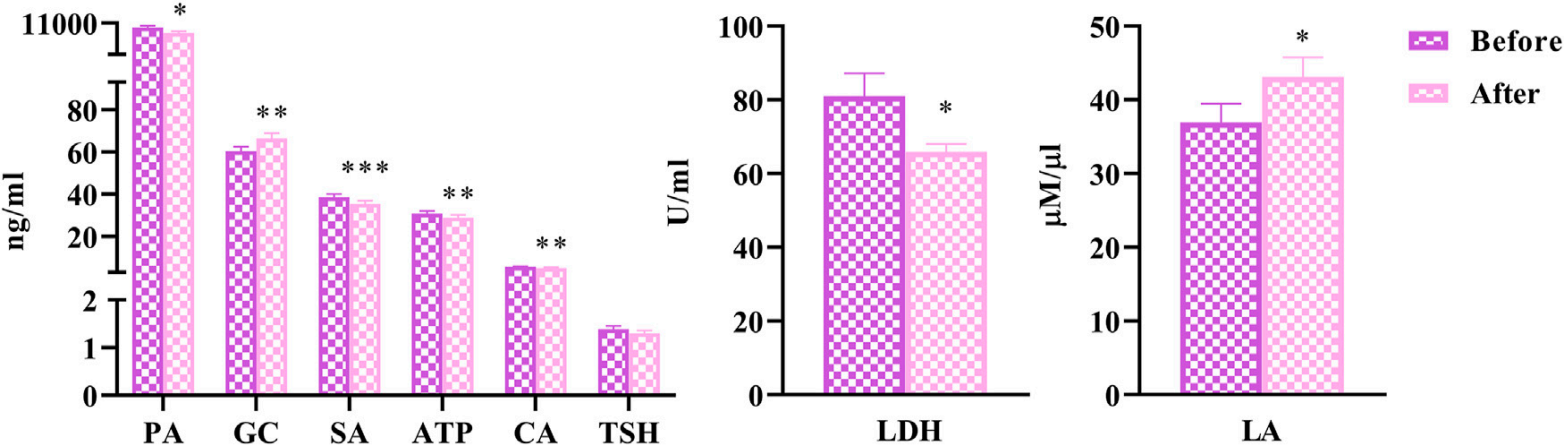
The changes of energy metabolism factors before and after HLJDD intervention (Before) Oral disease group (After) HLJDD intervention group (*p < 0.05, **p < 0.01, ***p < 0.001).

To explore the effects of HLJDD on energy metabolism in people with “Shanghuo”, we measured 7 energy metabolism factors before and after HLJDD intervention. Compared with the oral disease group, the HLJDD intervention group showed significantly reduced levels of SA (*p* < 0.001), ATP, CA (*p* < 0.01), LDH and PA (*p* < 0.05) after HLJDD intervention. The levels of GC (*p* < 0.01) and LA (*p* < 0.05) significantly increased and the level of TSH showed a downward trend after HLJDD intervention (Figure 2).

### Oxidative Stress

**FIGURE 3:**
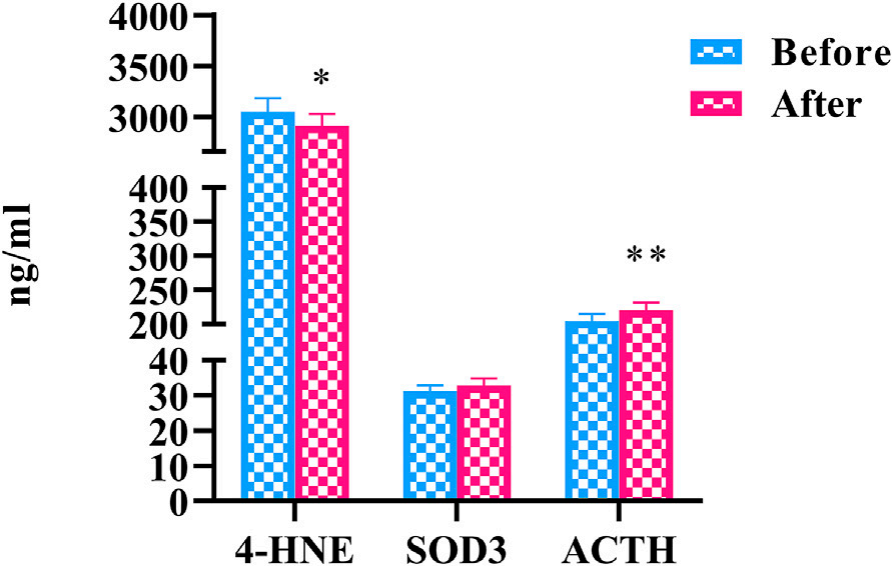
The changes of oxidative stress factors before and after HLJDD intervention (Before) Oral disease group (After) HLJDD intervention group (*p < 0.05, **p < 0.01).

To elucidate the effects of HLJDD on oxidative stress in people with “Shanghuo”, we measured 3 oxidative stress factors. Compared with the oral disease group, the HLJDD intervention group showed significantly reduced levels of 4-HNE (*p* < 0.05). The level of ACTH was significantly increased (*p* < 0.01) and the level of SOD3 showed a nonsignificant upward trend after HLJDD intervention (Figure 3).

### Huanglian Jiedu Decoction Regulated Differential Metabolites in“Shanghuo”Patients

**FIGURE 4:**
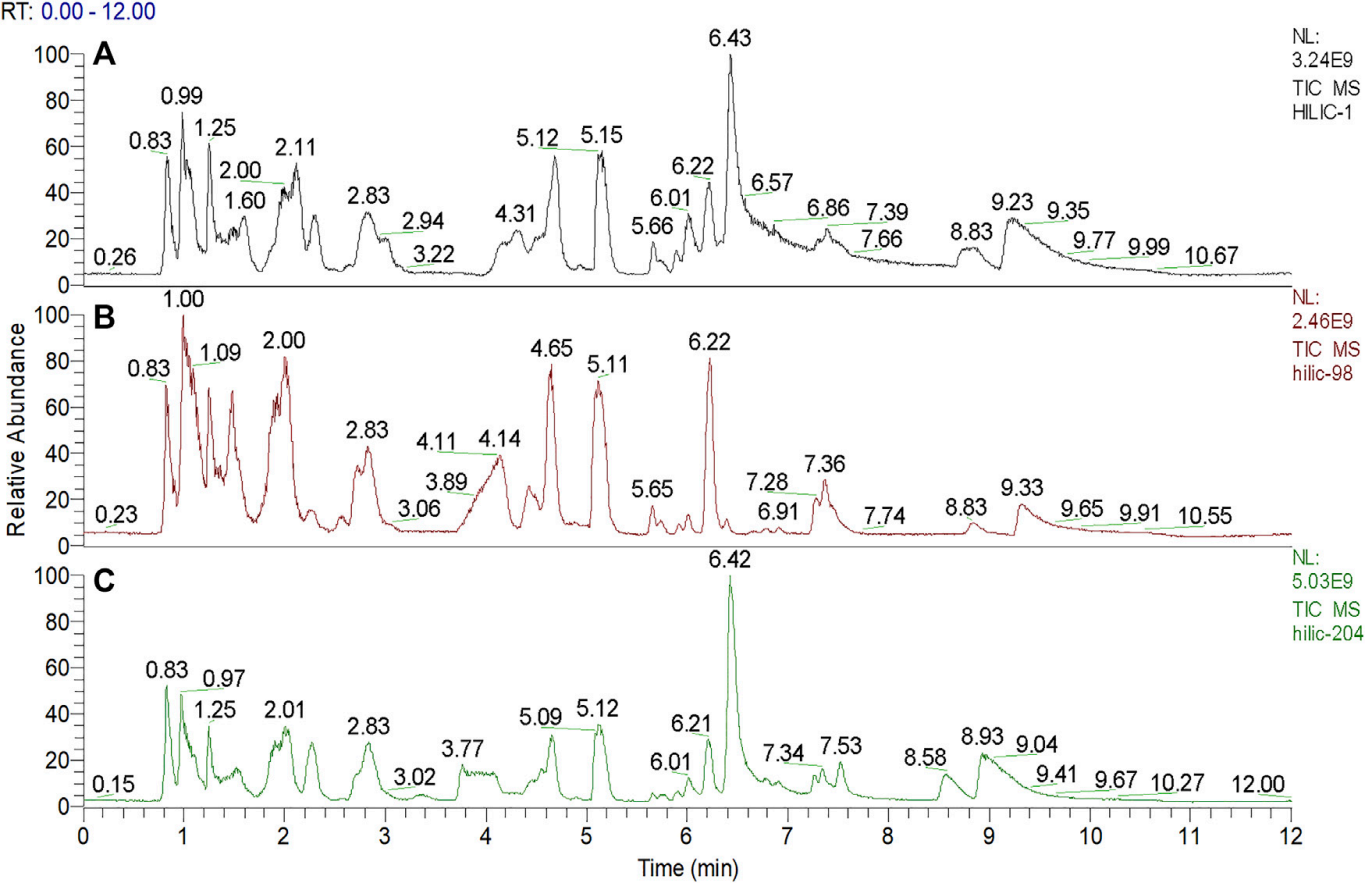
The total ion current chromatograms for ultra-high performance liquid chromatography-mass spectrometric analysis deriving from three groups. **(A)** Oral disease group **(B)** HLJDD intervention group **(C)** Healthy control group.

**FIGURE 5:**
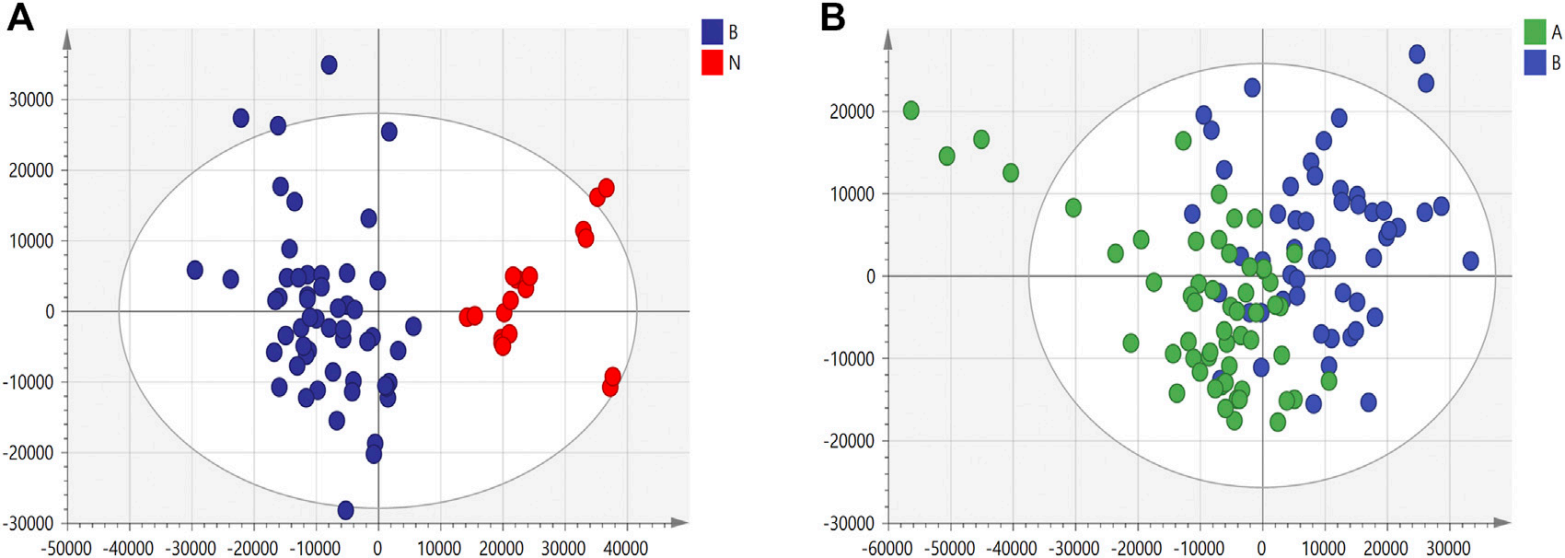
The PCA score plots under positive ion mode. **(A)** Healthy control group and Oral disease group **(B)** Oral disease group and HLJDD intervention group. Red dot represents the healthy control group, blue dot represents the oral disease group, green dot represents the HLJDD intervention group.

**FIGURE 6:**
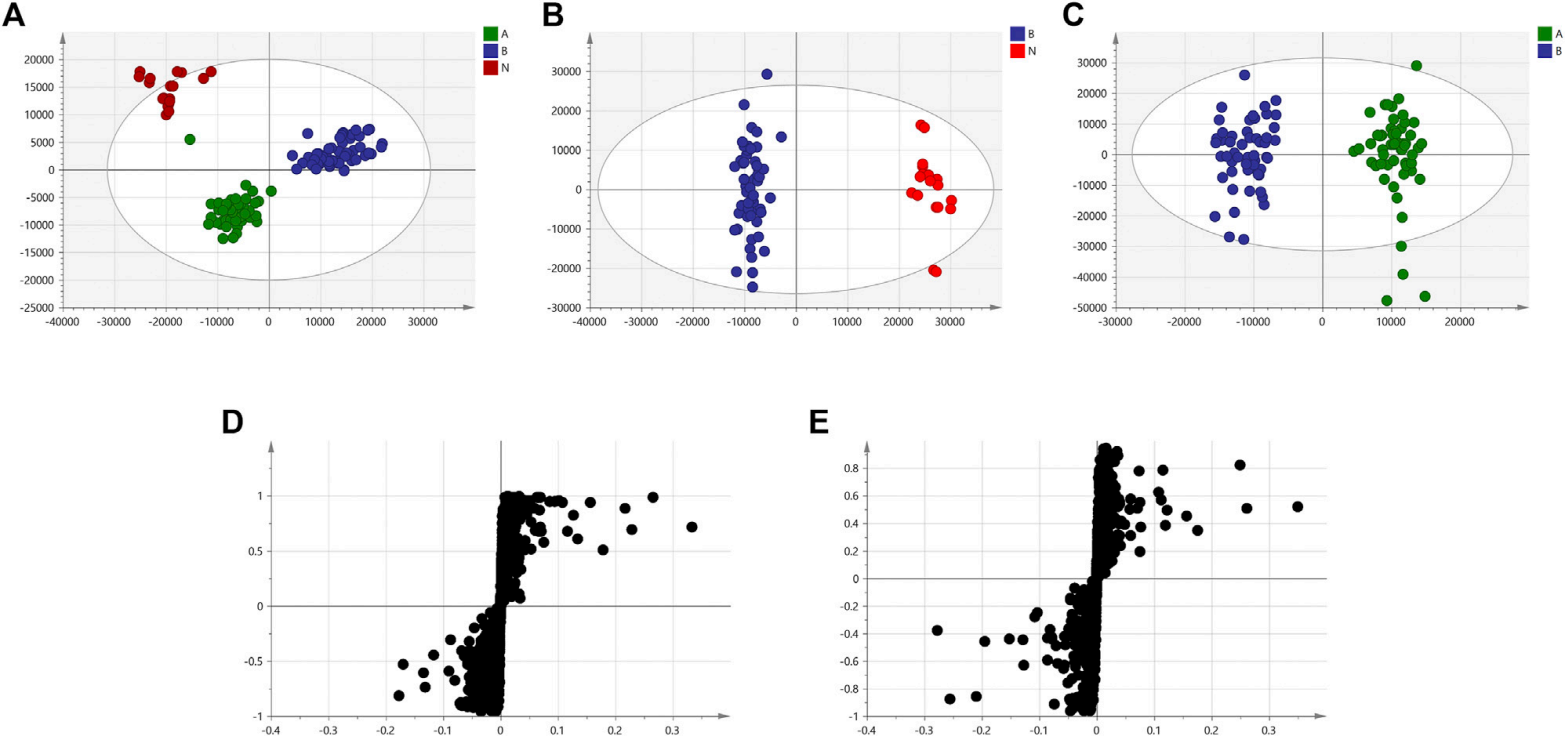
The OPLS-DA score plots and corresponding S-plot loadings plots under positive ion mode. **(A)** OPLS-DA score plot of the oral disease group, the HLJDD intervention group and the healthy control group **(B)** Healthy control group and Oral disease group **(C)** Oral disease group and HLJDD intervention group. Red dot represents the healthy control group, blue dot represents the oral disease group, green dot represents the HLJDD intervention group. **(D)** S-plot loadings plot of Healthy control group and Oral disease group **(E)** Oral disease group and HLJDD intervention group.

**FIGURE 7:**
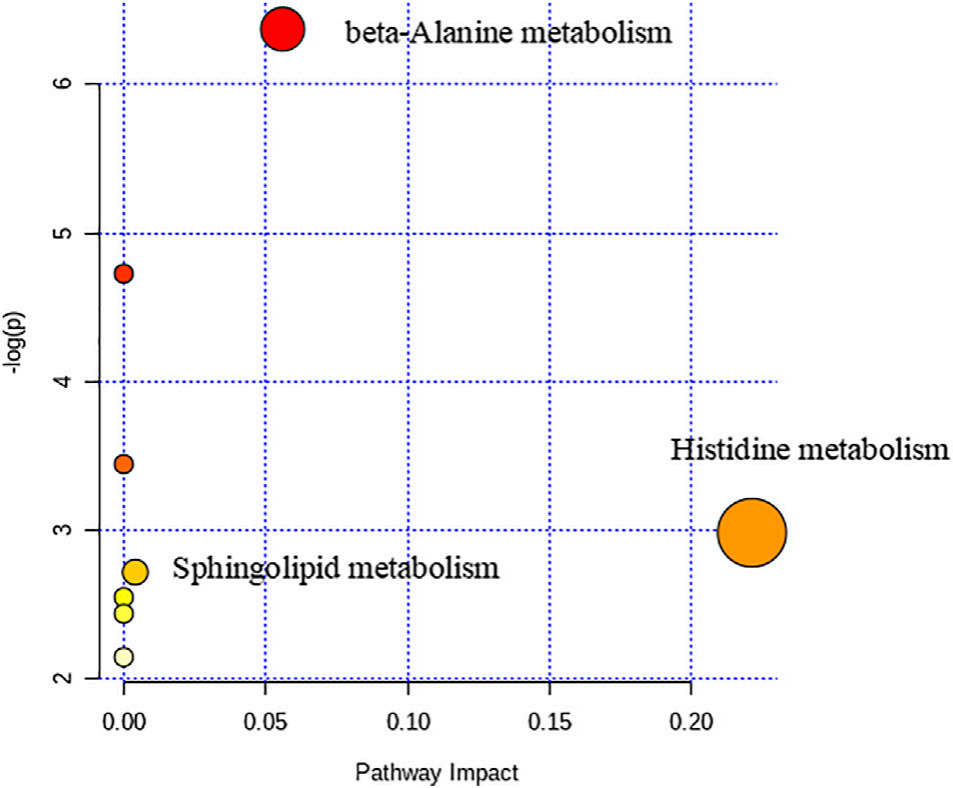
The Metabolic pathways of HLJDD interfered with “Shanghuo”.

We used nontargeted metabolomics to identify differential metabolites. For HILIC separation, the total ion chromatograms are shown in Figure 4. The chromatograms of the three groups we studied were significantly different, and further information was obtained using PCA and OPLS-DA. Figures 5, 6 show the PCA and OPLS-DA score plots between the different groups. The PCA score plot parameters obtained between the oral disease group and healthy control group, oral disease group and HLJDD intervention group were R2X = 0.774, Q2 = 0.374 and R2X = 0.797, Q2 = 0.312 respectively (Figures 5A,B). OPLS-DA was employed to maximize the differences between the groups and to aid in the identification of marker metabolites responsible for class separation. The parameters of the OPLS-DA score plot obtained from these three groups (Figure 6A) were R2X = 0.914, R2Y = 0.731, and Q2 = 0.867 in the positive mode. These values indicated that there were clear separations among the oral disease patient group, HLJDD group and healthy control group. The corresponding S-plot loading plots of OPLS-DA between each two groups are shown in Figures 6D,E. Each point in the S-plot loading plots represents the metabolite-related information in the samples. Seventeen differential metabolites were initially identified in the healthy control group and oral disease group (Table 1). Thirteen differential metabolites were preliminarily identified in the oral disease group and HLJDD intervention group (Table 2). There were 7 differential metabolites that were common among the three groups, and they had a certain improvement trend under the regulation of HLJDD. Among them, l-lysine, phytosphingosine and armillaripin were increased, and l-carnitine, l-histidine, spermine, and pyroglutamylglycine were reduced after HLJDD intervention. The 7 differential metabolites identified were imported into MetaboAnalyst software for analysis of metabolic pathways, and we found that HLJDD interfered with “Shanghuo” and mainly affected histidine metabolism, beta-alanine metabolism, sphingolipid metabolism pathways (Figure 7).

**TABLE 1:** Identification of biomarkers in the oral disease group and the healthy control group.

No	Compound	Element composition	Retention time (min)	Mass (Da)	Adducts	Mass error (ppm)	Trend
1	Betaine	C5H11NO2	4.65	118.0866	M + H	2.91	Up
2	l-carnitine	C7H15NO3	4.53	162.1126	M + H	0.94	Up
3	Creatine	C4H9N3O2	5.65	132.077	M + H	1.66	Down
4	l-Proline	C5H9NO2	7.45	116.071	M + H	3.67	Down
5	l-Histidine	C6H9N3O2	7.28	156.077	M + H, M + Na	1.47	Up
6	l-Lysine	C6H14N2O2	7.37	147.113	M + H, M + Na	1.13	Down
7	l-cystine	C6H12N2O4S2	7.55	241.0312	M + H	0.41	Up
8	l-acetylcarnitine	C9H17NO4	2.91	204.1232	M + H	0.87	Up
9	Ornithine	C5H12N2O2	7.45	133.0974	M + H	1.77	Down
10	Decanoylcarnitine	C17H33NO4	1.48	316.2483	M + H	0.15	Up
11	l-octanoylcarnitine	C15H29NO4	1.58	288.217	M + H	0.075	Up
12	Propionylcarnitine	C10H19NO4	2.38	218.1389	M + H	0.91	Down
13	Spermine	C10H26N4	9.04	203.2234	M + H	1.62	Up
14	Phytosphingosine	C18H39NO3	1.46	318.3003	M + H	0.21	Down
15	Armillaripin	C24H30O6	0.87	415.2116	M + H, M + Na	0.22	Down
16	Neurine	C5H13NO	2.71	104.1075	M + H	4.53	Down
17	Pyroglutamylglycine	C7H10N2O4	6.30	187.0716	M + H	1.17	Up

**TABLE 2:** Identification of biomarkers in the HLJDD intervention group and the oral disease group.

No	Compound	Element composition	Retention time (min)	Mass (Da)	Adducts	Mass error (ppm)	Trend
1	l-carnitine	C7H15NO3	4.53	162.1126	M + H	0.94	Down
2	l-Histidine	C6H9N3O2	7.28	156.0770	M + H, M + Na	1.47	Down
3	l-Lysine	C6H14N2O2	7.37	147.1130	M + H, M + Na	1.13	Up
4	Sphinganine	C18H39NO2	1.44	302.3055	M + H	0.32	Up
5	l-Arginine	C6H14N4O2	7.27	175.1192	M + H, M + Na	1.24	Up
6	Creatinine	C4H7N3O	2.83	114.0666	M + H, M + Na	3.21	Up
7	l-Glutamine	C5H10N2O3	6.19	147.0766	M + H	0.96	Down
8	Spermine	C10H26N4	9.04	203.2234	M + H	1.62	Down
9	Phytosphingosine	C18H39NO3	1.46	318.3003	M + H	0.21	Up
10	Alanyl-Serine	C6H12N2O4	7.53	177.0871	M + H, M + Na	0.88	Up
11	Armillaripin	C24H30O6	0.87	415.2116	M + H, M + Na	0.22	Up
12	N-Methylethanolaminium phosphate	C3H10NO4P	4.61	156.0423	M + H	1.67	Down
13	Pyroglutamylglycine	C7H10N2O4	6.30	187.0716	M + H	1.18	Down

### Huanglian Jiedu Decoction Regulated Distinct Lipid Profiles in“Shanghuo”Patients

**FIGURE 8:**
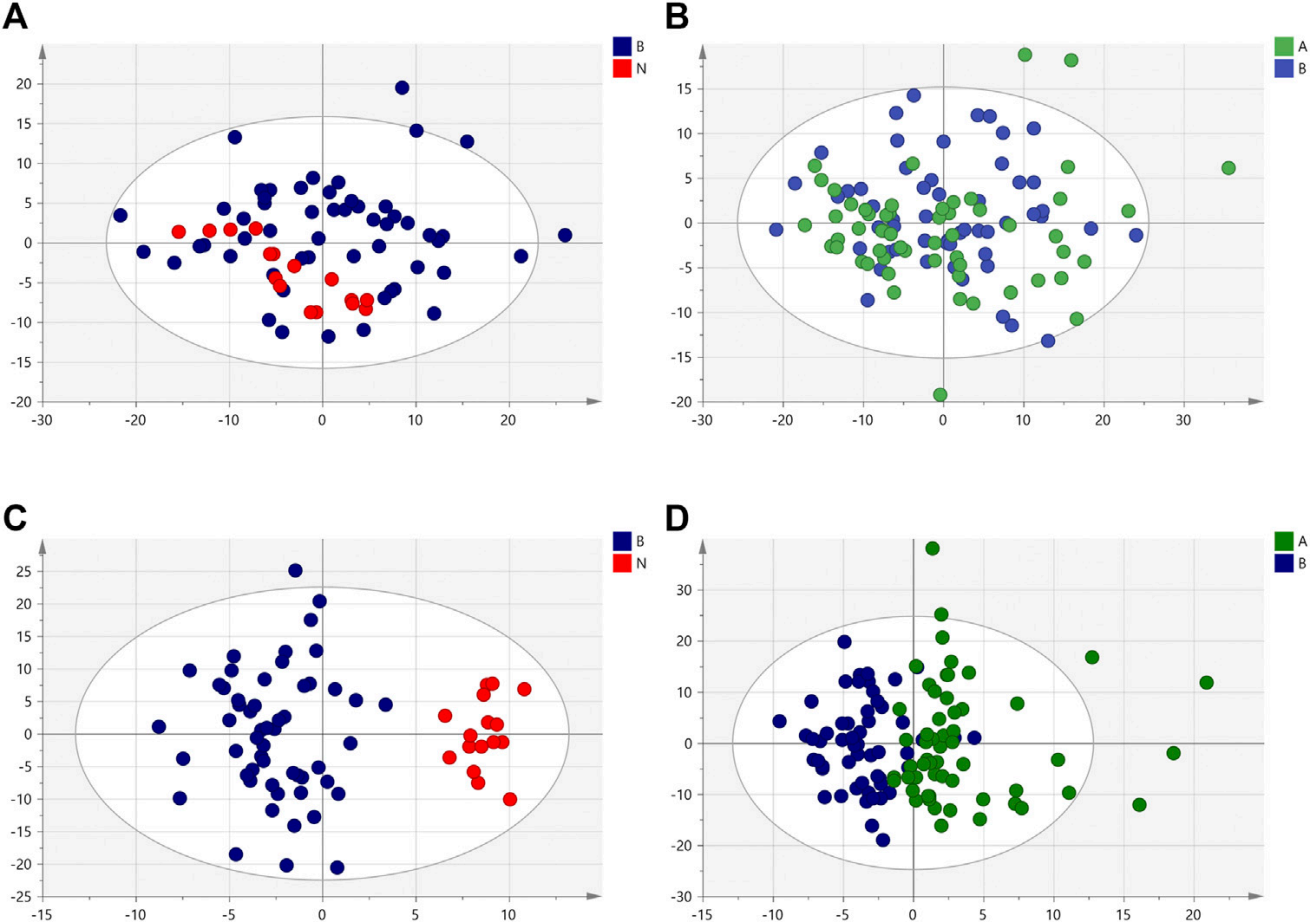
The PCA and OPLS-DA score plots of lipid sample under positive ion mode. **(A,C)** Healthy control group and Oral disease group **(B,D)** Oral disease group and HLJDD intervention group. Red dot represents the healthy control group, blue dot represents the oral disease group, green dot represents the HLJDD intervention group.

We used the targeted lipid metabolomics method to study the changes in lipid metabolites. Similar to the nontargeted metabolomics analysis method, PCA and OPLS-DA were performed on the plasma sample data. Figure 8 shows the PCA and OPLS-DA score plots among the oral disease group, HLJDD intervention group and healthy control group. The parameters of the PCA score plot obtained between the oral disease group and healthy control group, oral disease group and HLJDD intervention group (Figures 8A,B) were R2X = 0.899, Q2 = 0.558 and R2X = 0.916, Q2 = 0.65. Corresponded to the order of comparison between each of the above two groups, the OPLS-DA score plot parameters (Figures 8C,D) were R2X = 0.588, R2Y = 0.821, Q2 = 0.754 and R2X = 0.459, R2Y = 0.466, Q2 = 0.363.

**FIGURE 9:**
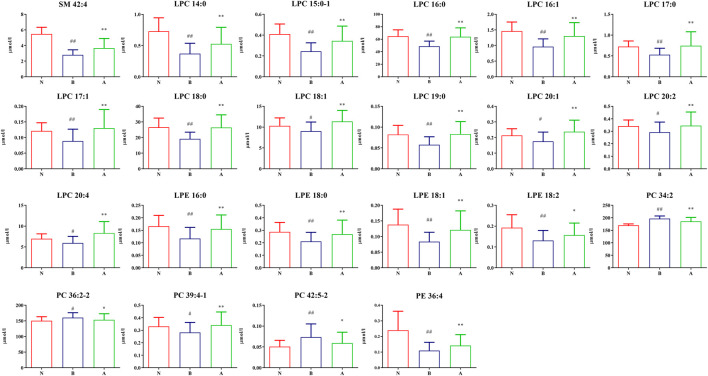
The differentially expressed lipid concentration of each group under positive ion mode. (N) Healthy control group; (B) Oral disease group; (A) HLJDD intervention group. Groups N and B (^#^ *p* < 0.05, ^##^ *p* < 0.01); Groups A and B (*p < 0.05, **p < 0.01).

**FIGURE 10:**
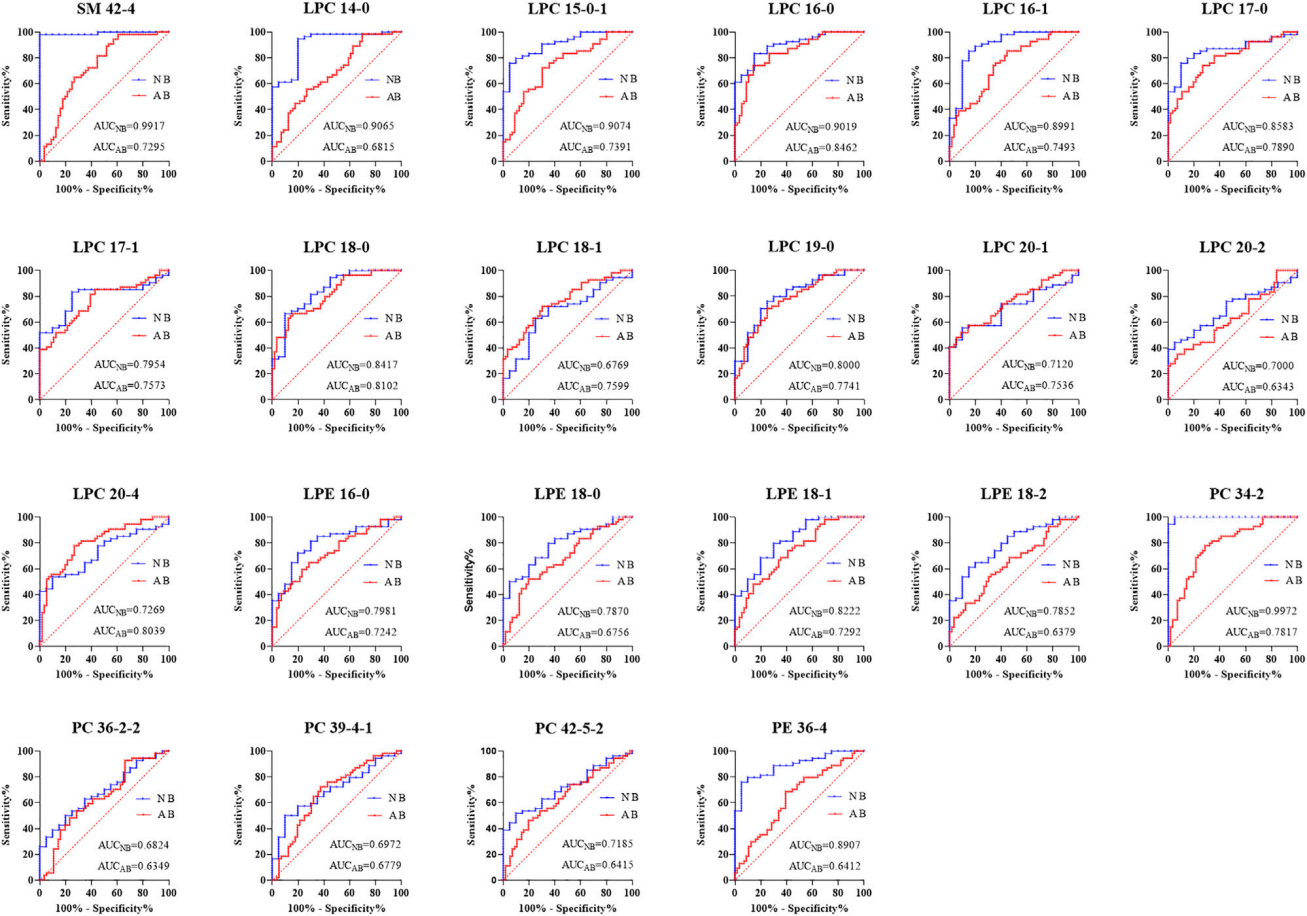
The discriminatory ability between the healthy control group and the oral disease group, the oral disease group and the HLJDD intervention group were analyzed using an ROC curve (NB) Healthy control group and Oral disease group (AB) Oral disease group and HLJDD intervention group.

We found the following 46 differential lipids in the healthy control group and oral disease group: lysophosphatidylcholine 14:0 (LPC 14:0), LPC 15:0–1, LPC 16:0, LPC 16:1, LPC 17:0, LPC 17:1, LPC 18:0, LPC 18:1, LPC 18:3, LPC 19:0, LPC 20:1, LPC 20:2, LPC 20:4, lysophosphatidylethanolamine 16:0 (LPE 16:0), LPE 18:0, LPE 18:1, LPE 18:2, phosphatidylcholine 26:0 (PC 26:0), PC 28:0, PC 32:2, PC 32:3, PC 34:2, PC 34:4, PC 36:2-2, PC 36:4-1, PC 36:6, PC 37:2-1, PC 37:3-1, PC 38:2-1, PC 39:4-1, PC 42:4, PC 42:5-2, phosphatidylethanolamine 34:1 (PE 34:1), PE 34:2, PE 34:3-2, PE 35:2, PE 36:1-2, PE 36:2-1, PE 36:4, PE 38:3-1, sphingomyelin 39:1 (SM 39:1), SM 40:3, SM 41:1, SM 42:4, SM 43:1, and SM 43:2. Furthermore, 26 differential lipids were discovered in the oral disease group and HLJDD intervention group. Moreover, 22 differential lipids were common among the three groups and had a certain improvement trend after HLJDD intervention. Among them, the concentrations of the following 19 lipids increased after HLJDD intervention: LPC 14:0, LPC 15:0–1, LPC 16:0, LPC 16:1, LPC 17:0, LPC 17:1, LPC 18:0, LPC 18:1, LPC 19:0, LPC 20:1, LPC 20:2, LPC 20:4, LPE 16:0, LPE 18:0, LPE 18:1, LPE 18:2, PC 39:4-1, PE 36:4 and SM 42:4. The concentrations of the following three lipids decreased after HLJDD intervention: PC 34:2, PC 36:2-2 and PC 42:5–2. The results indicated that the differential lipids were mainly concentrated in lysophosphatidylcholines (LPCs), lysophosphatidylethanolamines (LPEs), phosphatidylcholines (PCs). The concentrations (μmol/L) of the differential lipids in each group under positive ion mode are shown in Figure 9. To assess the discriminatory ability of the 22 aforementioned differential lipids between the healthy control group and oral disease group, and between the oral disease group and HLJDD intervention group, ROC analyses were applied to calculate the areas under the curve (AUCs) (Figure 10). Thirteen of the 22 differential lipids had the diagnostic value in distinguishing the plasma samples of the healthy control group and oral disease group and the samples of the oral disease group and HLJDD intervention group. The AUCs of these lipids, which included LPC 15:0–1, LPC 16:0, LPC 16:1, LPC 17:0, LPC 17:1, LPC 18:0, LPC 19:0, LPC 20:1, LPC 20:4, LPE 16:0, LPE 18:1, PC 34:2 and SM 42:4 were more than 0.7.

### Network Analysis of Differentially Expressed Biomarkers

**FIGURE 11:**
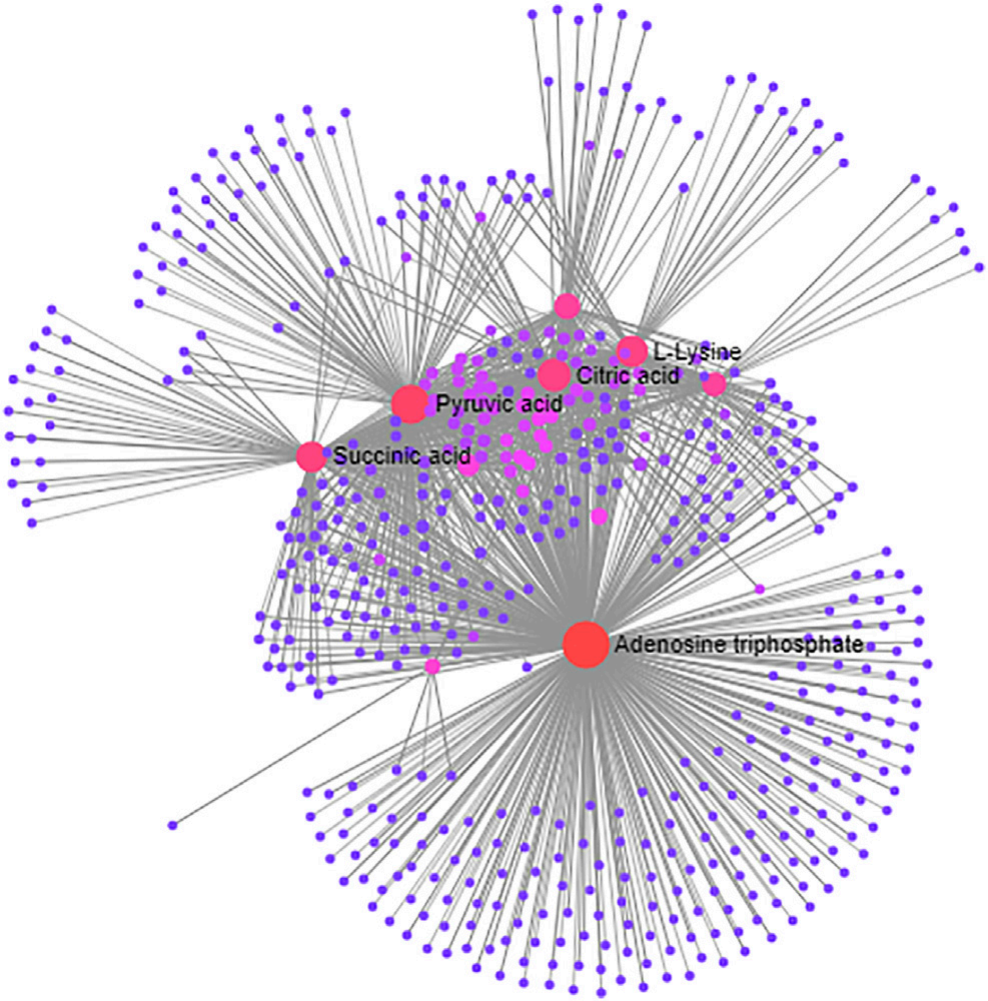
The KEGG global metabolic network analysis of differentially expressed biomarkers based on multiple influencing factors and multi-omics analysis.

**FIGURE 12:**
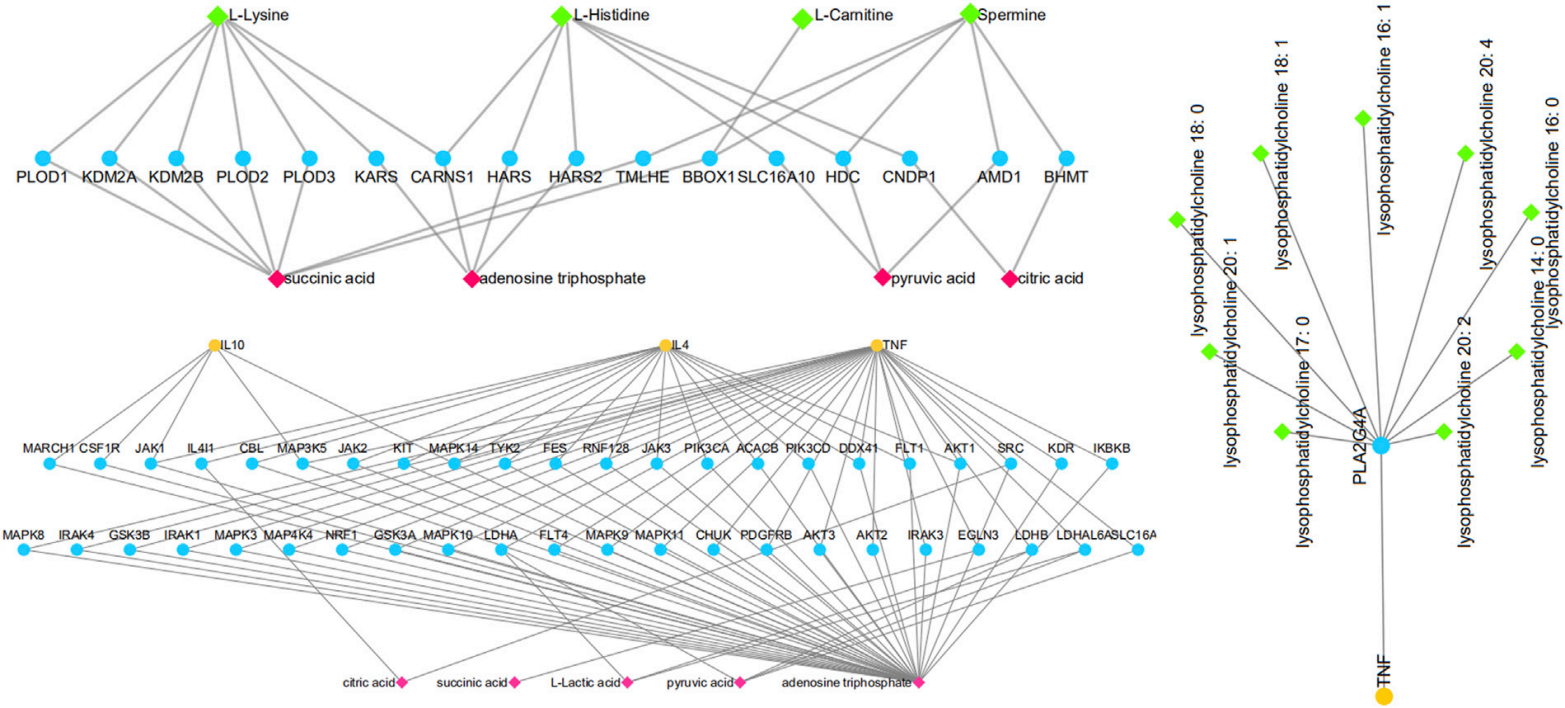
The protein-enzyme/transporter-metabolite network analysis of differentially expressed biomarkers based on multiple influencing factors and multi-omics analysis. Green represents different metabolites; Red represents energy metabolism factors; Yellow represents inflammatory factors; Blue represents metabolic enzyme/transporter.

The network associations of these differentially expressed biomarkers based on multiple influencing factors (inflammatory factors, oxidative stress and energy metabolism factors) and multi-omics analysis (targeted and nontargeted analysis) were explored through MetaboAnalyst and Cytoscape v3.4.0 software from three perspectives, including the KEGG global metabolic network, metabolite-metabolite interaction network and protein-enzyme/transporter-metabolite network. The potential pathways of differentially expressed biomarkers based on KEGG global metabolic network analysis are listed according to their *p*-values (*p* < 0.01) (Table 3). The results demonstrated that the differential biomarkers were closely related to the following pathways: the TCA cycle; alanine, aspartate and glutamate metabolism; pyruvate metabolism; and beta-alanine metabolism. From the aspect of metabolite-metabolite interaction network analysis, ATP was the key point (Figure 11). Furthermore, the relationships of differentially expressed biomarkers were clarified and built directly or indirectly through protein-enzyme/transporter-metabolite network analysis (Figure 12). The differential lipids and metabolites were correlated with some inflammatory factors, such as IL-10, IL-4, and TNF-α. And TNF-α influenced the differential lipids mainly through PLA2G4A. Moreover, ATP was observed to potentially interact with IL-10, TNF-α, and IL-4. Additionally, ATP prominently regulated differential metabolites, such as l-histidine, l-lysine. Furthermore, ATP was identified to possibly participate in the regulation of energy metabolism factors, including citric acid, succinic acid, and pyruvic acid.

**TABLE 3:** KEGG global metabolic network analysis of differential biomarkers.

Pathways	*p*-value
Citrate cycle (TCA cycle)	0.000118
Alanine, aspartate and glutamate metabolism	0.000662
Pyruvate metabolism	0.00667
Beta-alanine metabolism	0.00813

## Discussion

Huanglian Jiedu Decoction (HLJDD) is a classical TCM formula with heat-dissipating and detoxifying effects, which is comprised of *Rhizoma Coptidis*, *Radix Scutellariae*, *Cortex Phellodendri* and *Fructus Gardeniae* at a ratio of 3:2:2:3. The components in TCM prescription are complex, but not all of them has pharmacological activity. Therefore, the separation and identification of these pharmacodynamic components are of great significance. So we conduct the qualitative analysis of the HLJDD granules and the quantitative analysis of its 9 main active ingredients. A total of 87 major compounds are tentatively screened and characterized. From the results, we can see that it mainly contains three types of compounds, namely alkaloids (coptisine hydrochloride, epiberberine, berberine, palmatine, et al.), flavonoids (baicalein, baicalin, wogonoside, et al.) and iridoids (genipin 1-gentiobioside, geniposide, et al.). Many studies have manifested that alkaloids from *Rhizoma Coptidis* and *Cortex Phellodendri*, favonoids from *Radix Scutellariae* and iridoids from *Fructus Gardeniae* are three major active components in HLJDD, therefore they are regarded as markers for quality control of HLJDD (Zhu et al., 2012; Ren et al., 2016; Oshima et al., 2018). Although these four herbs of HLJDD have unique activities, they play a special role when combined. Compared with single herb or couplets, HLJDD tends to show higher C_max_, shorter T_max_ and better pharmacological effects (Pan et al., 2018). HLJDD has exhibited pharmacological activities in various aspects, including anti-tumer, anti-infammatory, anti-allergy, lipid-modulating, gut microbiota-modulating, anti-bacterial, and hepatoprotection (Qi et al., 2019). Our research focus on the influence of HLJDD on inflammation, oxidative stress factors and energy metabolism aspects in “Shanghuo”, and explore its intervention in other endogenous components through nontargeted and targeted lipid metabonomics analysis to find the differential metabolites in HLJDD to interfere with “Shanghuo” , and to lay the foundation for finding biomarkers in the future.

### Inflammatory Factors

In TCM, it is believed that endogenous and exogenous heat and toxins are the pathogenic mechanism of infammation. To some extent, infammatory and allergic mediators, as well as infammatory factors generated by infammations and allergies are considered as toxins leading to the heat syndromes to appear in the context of infammatory and allergic responses. HLJDD inhibits the inflammatory process by reducing the accumulation of reactive oxygen free radicals, interfering with lipid metabolism pathways, and regulating mitochondrial membrane potential. Analysis of potential molecular mechanisms and core targets showed that the NF-κB, STAT and PPAR pathways reduced the expression of inflammatory factors to achieve inflammatory immunity (Liu et al., 2019). In particular, the anti-inflammatory effect of HLJDD has been widely used to treat various diseases such as gastritis and aphthous stomatitis (Cao et al., 1996).

Clinical and experimental studies have confirmed that when oral ulcers occur, abnormally activated macrophages in the oral mucosa secrete a large number of proinflammatory factors, and the activated NF-κB inhibitory protein (IκB) kinase complex promotes NF-κB translocation to the nucleus, indirectly initiating the expression of genes such as the proinflammatory factors TNF-α and IL-1β, and inhibiting the expression of anti-inflammatory factors. The balance between anti-inflammatory factors and proinflammatory factors is disrupted, thereby exacerbating oral mucosal damage (Sun et al., 2013; Han et al., 2015; Bo et al., 2015). TNF-α has also been found to be significantly increased in the saliva of ROU patients, which might induce IL-2 and IL-6 (Hegde et al., 2018). In addition, IL-8 was more sensitive than IL-6 in humoral monitoring activities for ROU (Sun et al., 2004). After treatment, serum IL-8 and TNF-α levels were significantly reduced (Ding and Zhang, 2019). IL-10 is closely related to the occurrence and development of various oral mucosal diseases, such as ROU, oral lichen planus, Behcet’s disease, and pemphigus (Wang and Li, 2016). Due to low levels of IL-10, which functions to inhibit the release of cytokines, the inflammatory reaction might not be suppressed; hence, ROU might occur. Studies have indicated that T-cell immunity plays a role in the etiology of ROU. T-cell immunity reactions have been described as TH1 and TH2 reactions. It is believed that IL-10 initiates TH2 reactions (Avci et al., 2014). IL-10 typically stimulates epithelial proliferation in the healing process. Low levels of ROU might suggest a delay in epithelialization and prolongation of the duration of the lesions. As the occurrence of ROU diminished with advancing age, it was also of interest that whole blood assays revealed greater IL-10 production in elderly individuals than in younger controls (Cakman et al., 1996). ROU is a common oral condition with a major impact on quality of life. The condition is thought to be due to the overexpression of T helper-1 (Th1)-related cytokines. Since interleukin-4 (IL-4) and its receptor (IL-4Rα) are antagonistic to Th-1 pathways, polymorphisms in their genes may also be involved in the pathogenesis of aphthous stomatitis. Studies have found that certain polymorphisms in the IL4 gene might make individuals susceptible to ROU (Najafi et al., 2018). In the present results, HLJDD mainly regulated the inflammatory state of “Shanghuo” by affecting the levels of IL-4, IL-8, IL-10 and TNF-α. Among these factors, TNF-α showed a significant difference. This was the most obvious improvement in the inflammation indicators we tested, and it provided a scientific basis for us to further investigate biomarkers of “Shanghuo”.

Monocyte chemotactic protein-1 (MCP-1, also known as chemokine CCL2) is a monocyte chemokine with strong specificity that can promote the chemotaxis and adhesion of monocytes to damaged endothelial cells. Recent studies have shown that MCP-1 plays an important role in the healing process by quickly binding to CC chemokine receptor 2 and activating the inflammatory signaling pathway, resulting in irreversible damage and necrosis of mucosal cells and tissue (Martinelli-Kläy et al., 2014). Other literature has reported that the expression level of MCP-1 increases significantly during the period of active periodontal disease (Li, 2011). MCP-1 induces monocytes and lymphocytes to respond to inflammatory factor stimulation and promotes a continuous increase in immune damage (Yu et al., 2017). Based on our results, we know that the level of MCP-1 has a tendency to decrease after HLJDD intervention. These results showed that HLJDD might have an anti-inflammatory effect.

In summary, by exploring changes in inflammation indicators, we found that HLJDD had a good therapeutic effect on “Shanghuo”. The results showed that HLJDD mainly regulated the synthesis and secretion of TNF-α, IL-10, IL-8 and IL-4.

### Energy Metabolism

TCM corrects the rise and fall of Yin and Yang, and the change in cold and heat in the human body by regulating the metabolism of the body. “Shanghuo” belongs to the category of “heat syndrome” in TCM. Modern research has shown that heat syndromes are closely related to energy metabolism, especially thyroid function and sodium pump activity (Xie and Wang, 2013).

In the “Shanghuo” state, the accumulation of ATP in the human body cause the AMP/ATP ratio to decrease; then, the function of AMPK is inhibited, and the catabolic pathway is suppressed. On the other hand, the anabolic pathway that consumes ATP is activated to promote energy conversion and utilization (Bao et al., 2018). In rats with excessive heat syndrome, the activities of Na + -K + -ATP, Mg2+-ATP, Ca2+-ATP, and Ca2+-Mg2+-ATP were significantly increased, showing that ATP decomposition and heat generation were enhanced (Si, 2012). Some research results have indicated that all components of *Radix Scutellariae* inhibit substance and energy metabolism. The effects of aglycones and glycosides are similar to those of the whole components of the herb and are more obvious in the heat-syndrome rats (Chen et al., 2020). The role of SA has gradually expanded into the fields of immunity and cancer. Increased SA content could further promote the stabilization of hypoxia-inducible factor-1α (HIF-1α) (Tannahill et al., 2013). In the synovium of rheumatoid arthritis, the content of HIF-1α is increased, which is crucial for IL-1β. The reason is that the continuous induction of the proinflammatory cytokine IL-1β requires HIF-1α. When SA and antigens were used to stimulate dendritic cells (DCs) at the same time, the activation of antigen-specific T cells and the production of TNF-α and IFN-γ were increased (Rubic et al., 2008). In our study, SA and ATP decreased significantly after HLJDD intervention (*p* < 0.001 and *p* < 0.01). Combined with the above literature research, we inferred that HLJDD might regulate energy metabolism, thereby mediating inflammatory factors to treat “Shanghuo”.

The TCA cycle is a common pathway for the completely oxidative decomposition of various nutrients in the body. Citrate synthase catalyzes the production of CA from acetyl-CoA and oxaloacetate at this stage (Alves et al., 2015). Studies have reported that the serum citrate synthase enzyme activity of the heat syndrome model is significantly increased (Zhao et al., 2017), indicating that the production of CA is increased. Our experimental research showed that the contents of CA and SA in human plasma decreased after HLJDD intervention, suggesting that HLJDD might have a certain inhibitory effect on the TCA cycle. PA is the final product of the glycolytic pathway. It is reduced to lactic acid for energy supply in the cytoplasm. The reversible transfer of hydrogen ions requires the coenzyme pyridine diphosphate (NAD^+^ or NADH). Under hypoxic conditions, PA is converted to LA by the action of LDH and NADH (Kirby et al., 2007). According to the literature, the content of LA and PA in plasma and the LDH activity of excess heat syndrome were significantly reduced after administering chloroform extract and acetic acid extract of *Rhizoma Coptidis* (Liu et al., 2012). Therefore, we could infer that HLJDD might treat “Shanghuo” by intervening in the glycolytic pathway and TCA cycle.

Thyroid-stimulating hormone (TSH) is a glycoprotein hormone synthesized and secreted by the pituitary gland that directly regulates thyroid function and controls the process leading to increased thyroid hormone production and secretion. It is a sensitive indicator of hypothalamic-pituitary-thyroid (HPT) axis function. Currently, research on TSH promoting the secretion of proinflammatory factors by adipocytes has mainly focused on subclinical hypothyroidism and cardiovascular diseases. Studies have found that the risk of hypercholesterolemia and cardiovascular disease increased among subclinical hypothyroidism patients, whose TSH levels were increased (Delitala et al., 2017). The expression of TSH increased in an animal model with real heat (Zhao et al., 2018). Our results showed that TSH showed a downward trend after HLJDD intervention in patients with “Shanghuo”. The results of these two experiments were complementary. Glucocorticoids (GCs) were important physiological substances in the body that can exert anti-inflammatory effects on macrophages by inhibiting their differentiation to an M1 phenotype (Xie et al., 2019). Consistent with our experimental results, the level of GC significantly increased after HLJDD intervention. We could infer that HLJDD might have glucocorticoid-related effects that prevented disease progression and deterioration in the process of energy metabolism during “Shanghuo”.

In conclusion, the effects of HLJDD intervention on energy metabolism in “Shanghuo” might mainly be related to the TCA cycle and glycolytic pathway.

### Oxidative Stress

Under normal circumstances, the production and elimination of ROS in the body maintain an oxidation-antioxidation balance. Once the balance is disrupted, the body develops oxidative stress, which causes damage to the mitochondrial respiratory chain. When “Shanghuo” occurs, the body is in a state of conflict between peroxidation and antioxidants. The increase in lipid peroxides indicates that a peroxidation reaction has occurred, and some symptoms of “Shanghuo” (such as oral ulcers and swollen gums) follow. Simultaneously, the antioxidant defense system is activated, and the total antioxidant level of T-AOC is increased. A large amount of glutathione and superoxide dismutases (SODs) are generated; therefore, the adverse stimulus is eliminated in time, which is one of the reasons for the low level of ROS in the body (Li et al., 2017). The antioxidative stress mechanism of HLJDD is closely related to the action of scavenging free radicals. The targets of the ingredients of HLJDD could affect the synthase that produces free radicals through cytokines. The mechanisms overlap with each other and enhance their antioxidant effects (Li et al., 2015).

SODs are enzymes that catalyze the removal of superoxide free radicals. In mammals, SOD1 (Cu/ZnSOD), SOD2 (MnSOD) and SOD3 (ecSOD) are three distinct members of this metalloenzyme family. SODs have been increasingly recognized for their regulatory functions in growth, metabolism and oxidative stress responses (Che et al., 2016). As an extracellular SOD enzyme, SOD3 not only acts as a passive antioxidant but also plays an active role in modulating redox signaling to support biological responses (Hu et al., 2019). Proteomics analysis has found that SOD3 expression is significantly reduced in individuals with “Shanghuo” compared to healthy individuals (Chen et al., 2017). Our study showed that there was a trend toward an increase in SOD3 after HLJDD intervention in “Shanghuo”, which echoes the discussion above.

Excessive oxygen free radicals induced oxidative stress reactions, which significantly increases lipid peroxidation products, particularly 4-hydroxynonenal (4-HNE). Under normal circumstances, 4-HNE is maintained at a very low physiological concentration in cells or tissues. When the body is stimulated and oxidative stress occurs, 4-HNE significantly increases, which has been confirmed in tissues such as hepatocytes, renal tubular cells, heart and small intestine (Blasig et al., 1995). In addition, a study found that 4-HNE gradually increased with age, and the concentration in patients with mild cognitive impairment was significantly increased compared with that in healthy people of the same age (Williams et al., 2006). 4-HNE is the most representative aldehyde product during lipid peroxidation and significantly inhibits cellular AMPK phosphorylation levels and induces cell damage. 4-HNE is mainly derived from the process of lipid peroxidation between fatty acid side chains and free radicals. Accelerating the oxidative metabolism of fatty acids and reducing the level of fatty acids in cells can effectively inhibit the production of 4-HNE (Xiao et al., 2017). Peroxisome proliferator-activated receptor *α* (PPARα) specifically binds to the peroxisome proliferator response element (PPRE) on the promoter of the gene encoding the key 4-HNE metabolic enzyme fatty aldehyde dehydrogenase (FALDH) gene, thereby upregulating FALDH expression, which accelerates the clearance of 4-HNE (Ronis et al., 2015). Tan IIA activates PPARα, promotes fatty acid metabolism in liver cells, and reduces the production of 4-HNE and 4-HNE protein adducts. In addition, TanⅡA also promotes the binding of PPARα and PPRE, accelerating the metabolic clearance of 4-HNE (Qian et al., 2019). When 4-HNE accumulates to a certain concentration, it causes toxicity, gene mutagenicity and carcinogenesis in cells (Shoeb et al., 2014). In our study, HLJDD reduced the increase in 4-HNE caused by “Shanghuo”. During stress, the most basic manifestation is a series of neuroendocrine changes, of which the dominant is strong stimulation of the hypothalamus-pituitary-adrenal cortex axis, which can be detected by ACTH levels. Compared with that in the oral disease group, the level of ACTH in the HLJDD intervention group was significantly increased in our experiment. The intervention mechanism still needs further study. In summary, the treatment of “Shanghuo” under oxidative stress conditions by HLJDD was related to changes in the levels of 4-HNE, SOD3 and ACTH.

### Huanglian Jiedu Decoction Regulated Differential Metabolites in“Shanghuo”Patients

Our results showed that l-lysine increased and l-carnitine decreased in the HLJDD intervention group compared with the oral disease group. l-lysine regulates cell proliferation, differentiation and excitability as well as intercellular interactions through the neurotransmitter system (Severyanova et al., 2019). The l-lysine diet enhances antioxidant activity by inhibiting the release of the inflammatory cytokine IL-6 (Zhu et al., 2017). To a certain extent, changes in the type or level of carnitine reflect the disturbance of fatty acid or amino acid metabolism in the body. Furthermore, elevated amino acid levels are caused by mitochondrial lipid overload and incomplete oxidation of fatty acids (Wang, 2016), which demonstrates that the disruption of amino acid metabolism might cause “Shanghuo”. These findings revealed that HLJDD regulates the metabolism of fatty acids or amino acids in the body to alleviate “Shanghuo”.

### Huanglian Jiedu Decoction Regulated Distinct Lipid Profiles in“Shanghuo”Patients

According to the classic TCM theory, the pathogenesis of metabolic syndrome was caused by excessive “heat” dissipation of body fluids. In addition, excessive lipid may lead to the accumulation of “heat”, which was eventually converted into toxins, a more serious cause (Qi et al., 2019). Also, lipids are important mediators of inflammation and play key roles in the development of various diseases, such as rheumatoid arthritis and atherosclerosis (Fuchs et al., 2005; Summerhill et al., 2019). LPE functions in intercellular signaling and in the activation of signaling enzymes and has been suggested to act through putative G protein-coupled receptors (Park et al., 2007). LPC increases the expression of adhesion molecules by promoting the phagocytosis of macrophages, monocytes and T lymphocytes, thereby exerting immunomodulatory effects, promoting cytoskeletal changes and regulating cell adhesion and migration capabilities (Kabarowski et al., 2002). The levels of plasma LPCs have been observed to decreased in sepsis patients, and one possible mechanism for the decrease in plasma LPC levels has been suggested to be enhanced conversion to LPA (Drobnik et al., 2003). Plasma levels of both total PC and total LPA were increased in experimental sepsis, suggesting that conversion from PC to LPC was decreased, whereas that from LPC to LPA was increased (Ahn et al., 2017). Our experimental results showed that all 12 differential lipids of LPCs were increased and three-quarters of the differential *p*Cs were decreased after HLJDD intervention. This change induced by HLJDD might be related to the promotion of the conversion of PC to LPC.

### Network Analysis of Differential Biomarkers

Some scholars found that the ATP content in the peripheral blood was significantly increased in the “Shanghuo” group (Bao et al., 2018), which corresponded to our research result that HLJDD reduced the ATP content of patients with “Shanghuo”. SA plays a key role in the process of ATP formation in the mitochondria and is an intermediate metabolite in the TCA cycle (Fan, 2018). Increased levels of metabolic intermediates in the TCA cycle, such as SA, CA, and malic acid, in “Shanghuo” population reflected that their energy metabolism was in a relatively vigorous state (Zhou et al., 2017). Mounting evidence indicates that ATP, namely, energy metabolism, is involved in the mechanism of “Shanghuo” and the therapeutic effect of HLJDD. In addition, TNF-α mainly affected differential lipids through PLA2G4A. It was reported in the literature that the long noncoding RNA SNHG14 promoted microglial activation by regulating miR-145–5p/PLA2G4A in cerebral infarction, and the miR-145–5p mimic reversed the increase in PLA2G4A and reduced the high levels of TNF-α and NO in BV-2 cells induced by SNHG14 overexpression (Qi et al., 2017). In summary, the overall results suggest that the differential biomarkers were closely correlated with ATP and the TCA cycle. We will validate these potential biomarkers and pathways and conduct in-depth mechanistic research in further phase II clinical studies of HLJDD.

## Conclusion

In the present clinical investigation, biochemical indicators of “Shanghuo” patients and the HLJDD intervention group were detected. We used multiplex cytokine assay technology to analyze inflammatory factors. ELISA technology was adopted to explore oxidative stress factors and energy metabolism factors. High Resolution-MS/MS technology was used comprehensively for nontargeted metabolomics and target lipidomics to elucidate the potential biomarkers closely related to the essence of “Shanghuo” and the treatment mechanism of HLJDD from the perspectives of the healthy control group, oral disease group and HLJDD intervention group. The results demonstrated that HLJDD regulated differentially expressed inflammatory factors (TNF-α, IL-10, IL-8 and IL-4), oxidative stress factors (4-HNE and ACTH), energy metabolism factors (CA, SA, GC, ATP, LDH, PA, and LA), and differential lipids and metabolites related to energy metabolism. Additionally, the network associations of those differentially expressed biomarkers were explored. All these differential biomarkers indicated that the mechanism of the therapeutic effect of HLJDD on “Shanghuo” mainly involved in the regulation of ATP and the TCA cycle. These findings are important for providing an investigational basis for understanding the pathophysiology of “Shanghuo” onset and the treatment mechanism of HLJDD. However, this study still has some limitations, multi-factor detection and multi-omics analysis only provide us with a direction, and in-depth research needs to continue. In the following research, we will focus on energy metabolism and the TCA cycle, study the changes in the content of key intermediates of the cycle in body fluids, and establish the cell model to detect this change process to further identify biomarkers and verify the intervention of HLJDD on the syndrome of “Shanghuo”.

## Ethics Approval and Consent to Participation

The Trial Registration Number: NCT03469232. Written informed consent was obtained from all patients. The study was conducted in accordance with the ethical principles of the Declaration of Helsinki, Good Clinical Practice implemented by the China Food and Drug Administration of the People's Republic of China, and International Ethical Guidelines Biomedical Research Involving Human Subjects promulgated by the Council for International Organizations of Medical Sciences. The clinical study protocol and informed consent forms were reviewed and approved by the Ethics Committee of Institute of Basic Research in Clinical Medicine (China Academy of Chinese Medical Sciences). All subjects were informed in oral and written forms of the objectives, procedures, and risks of participating in the study.

## Data Availability

The raw data supporting the conclusions of this article will be made available by the authors, without undue reservation.

## Glossary

HLJDD: Huanglian Jiedu Decoction
IL-4: interleukin-4
IL-1β: interleukin-1beta
IL-6: interleukin-6
IL-8: interleukin-8
IL-10: interleukin-10
IL-12p70: interleukin-12p70
IL-13: interleukin-13
MCP-1: monocyte chemotactic protein-1
MIP-1α: macrophage inflammatory protein-1alpha
TNF-α: tumor necrosis factor-alpha
CA: citric acid
SA: succinic acid
GC: glucocorticoid
ATP: adenosine triphosphate
LDH: lactate dehydrogenase
LA: lactic acid
PA: pyruvic acid
TSH: Thyroid-stimulating hormone
SOD3: extracellular superoxide dismutase
4-HNE: 4-hydroxynonenal
ACTH: adrenocorticotropic hormone;
TCM: traditional Chinese medicine
TCA: tricarboxylic acid
LPCs: lysophosphatidylcholines
LPEs: lysophosphatidylethanolamines
PCs: phosphatidylcholines
SMs: sphingomyelins
ROC curve: receiver operating characteristic curve
AUC: area under the curve
PCA: principal component analysis
OPLS-DA: orthogonal partial least squares discrimination analysis
MS: mass spectrum
ROU: recurrent oral ulceration
ROS: reactive oxygen species
